# Affinity maturation of Cry1Aa toxin to the *Bombyx mori* cadherin-like receptor by directed evolution based on phage display and biopanning selections of domain II loop 2 mutant toxins

**DOI:** 10.1002/mbo3.188

**Published:** 2014-07-16

**Authors:** Haruka Endo, Yuki Kobayashi, Yasushi Hoshino, Shiho Tanaka, Shingo Kikuta, Hiroko Tabunoki, Ryoichi Sato

**Affiliations:** 1 Graduate School of Bio-Applications and Systems Engineering, Tokyo University of Agriculture and TechnologyKoganei, Tokyo, 184-8588, Japan; 2 Department of Biological Production, Faculty of Agriculture, Tokyo University of Agriculture and TechnologySaiwai-cho 3-5-8, Fuchu, Tokyo, 183-8509, Japan

**Keywords:** Biopanning, cadherin-like receptor, Cry toxin, directed evolution, phage display

## Abstract

Directed evolution of a Cry1Aa toxin using phage display and biopanning was performed to generate an increased binding affinity to the *Bombyx mori* cadherin-like receptor (BtR175). Three mutant toxins (_371_WGLA_374_, _371_WPHH_374_, _371_WRPQ_374_25) with 16-, 16-, and 50-fold higher binding affinities, respectively, for BtR175 were selected from a phage library containing toxins with mutations in domain II loop 2. However, the observed toxicities of the three mutants against *B. mori* larvae and cultured cells expressing the BtR175 toxin-binding region did not increase, suggesting that increased binding affinity to cadherins does not contribute to the insecticidal activity. Affinity maturation of a Cry toxin to a receptor via directed evolution was relatively simple to achieve, and seems to have potential for generating a toxin with increased insecticidal activity.

## Introduction

The bacterial insect pathogen *Bacillus thuringiensis* (*Bt*) is used for the biological control of insect pests (Sanchis and Bourguet 2008) and disease vectors (Boyce et al. 2013). *Bt* produces crystalline inclusions of insecticidal proteins called Cry toxins during sporulation. After ingestion, the toxin is solubilized and partly digested by the alkaline midgut digestive fluids of insects to form an active toxin core. The activated toxin then diffuses through the peritrophic membrane and specifically interacts with receptors on the lumen side of midgut epithelial cells, resulting in cell lysis, disintegration of midgut epithelial tissue, and death of the insect (Pigott and Ellar 2007). The Cry toxins naturally produced by *Bt* are highly selective for target insects. The insecticidal spectrum is narrow and the insecticidal activities are low in some susceptible insects. As collecting promising *Bt* strains from the soil is not always a simple task, establishing a protein engineering method for the generation of Cry toxins with higher activities and wider insecticidal spectra is required to make Cry toxins more suitable for industrial applications.

The mode of action of Cry toxins has been widely studied. In particular, research on the activity of Cry1A toxins has resulted in two representative models. In the pore-forming model, the toxin binds a cadherin-like receptor and forms oligomers that are believed to insert into the cell membrane after binding glycosylphosphatidylinositol-anchored receptors such as aminopeptidase N (APN) and alkaline phosphatase (ALP) (Bravo et al. 2011). This induces pore formation and kills the enterocyte by osmotic cell lysis (Bravo et al. 2011). The signal-transduction model suggests that the binding of Cry toxin monomers to a cadherin-like receptor induces programed cell death by activating the adenylyl cyclase/PKA-signaling pathway (Zhang et al. 2005). Both models indicate that the cadherin-like receptor has an important role in the mode of action of Cry1A. In fact, many Cry1A-resistant strains have mutations in their cadherin-like receptors (Morin et al. 2003; Yang et al. 2009; Gahan et al. 2010). Ectopic expression of cadherin-like receptors from insects that are susceptible to Cry1A resulted in cultured cells with susceptibility to the toxin (Nagamatsu et al. 1999; Tsuda et al. 2003; Hua et al. 2004; Flannagan et al. 2005; Zhang et al. 2005; Jurat-Fuentes and Adang 2006). These studies also support the functional importance of the cadherin-like receptor. Recently, a new hypothesis was reported in which ABC transporter family C2 and cadherin-like receptors synergistically function as receptors during the induction of osmotic cell lysis (Tanaka et al. 2013).

Cry toxins are composed of three conserved domains (Li et al. 1991). Domain I, the N-terminus domain, contains a seven *α*-helix bundle and is involved in cytotoxicity (Li et al. 1991). Domain II, the middle domain, is a *β*-prism that is implicated in receptor interactions and insect specificity (Rajamohan et al. 1996; Wu and Dean 1996; Gómez et al. 2003; Fernández et al. 2008). Domain III, the C-terminus domain, is a *β*-sandwich that affects receptor interactions, insect specificity (Schnepf et al. 1998; de Maagd et al. 2003), and stabilization of molecular structure (Grochulski et al. 1995). Cry toxins are thought to bind cadherin-like receptors via loop *α*8 (Gómez et al. 2003), loop 1 (Wu and Dean 1996), loop 2 (Gómez et al. 2001), and loop 3 (Xie et al. 2005) of domain II. Research focusing on the protein engineering of these regions has generated drastic improvements in insecticidal activity and spectrum. Amino acid substitution of Cry4Ba domain II loop 3 resulted in to 700- and 285-fold higher insecticidal activities in *Culex quinquefasciatus* and *Culex pipiens*, respectively (Abdullah et al. 2003). Cry1Aa, normally active against lepidopteran insects, was changed in specificity to the mosquito *C. pipiens*, when a mutation was introduced in loops 1 and 2 of domain II (Liu and Dean 2006). However, in many combinations of Cry toxin and insect pest, the regions on the Cry toxin that should be modified are not clear. Furthermore, a high-throughput screening method for an improved Cry toxin resulting from protein engineering has not yet been established.

Note that since receptor interactions are involved in insect specificity and toxicity (Pigott and Ellar 2007), a mutant toxin with a higher binding affinity to a receptor, such as the cadherin-like receptor, might exhibit a higher toxicity. Hence, screening of affinity-maturated mutants to cadherin-like receptors may allow for high-throughput screening of activity-enhanced Cry toxins. The T7 phage libraries, which contain up to 10^6^ variations of Cry1Aa toxins, were generated by introducing random, tetrameric amino acid substitutions in the *Bombyx mori* cadherin-like receptor (BtR175)-binding region of the toxin (Fujii et al. 2013). Subsequently, biopanning was used as a high-throughput screening method for evolutionary molecular engineering (Fujii et al. 2013) to select phage clones displaying mutant toxins with a high binding affinity for BtR175. Three mutant toxins with 13-, 15-, and 42-fold higher affinities were successfully acquired from the loop 3 mutant toxin library (Fujii et al. 2013). However, despite the enhanced binding affinities, the toxicities of these mutants against insect individuals or Sf9 cells expressing the BtR175-toxin-binding-region (BtR175-TBR) did not increase (Fujii et al. 2013). The introduced mutations are thought to hinder the mode of toxicity of Cry1Aa outside of the BtR175 interaction. For example, since domain II loop 3 is a putative APN-binding region (Gómez et al. 2006; Pacheco et al. 2009), the binding affinity of Cry1Aa to APN may have decreased.

This report describes the selection of affinity-maturated mutant Cry1Aa toxins from the loop 2 library. Since loop 2 is not in the putative APN-binding region, a loop 2 mutation might not affect any of the phases of the mode of action of Cry1Aa. In addition, loop 2 has been reported as a cadherin-like binding region. Affinity-maturated mutant toxins with 16-, 16-, and 50-fold higher binding affinities to BtR175 were obtained. This indicates that loop 2 is a suitable region for introducing mutations that induce affinity maturation of Cry1Aa to BtR175. However, the affinity-maturated mutants showed no substantial enhancements in insecticidal activity. This report discusses the remaining possible strategy for BtR175-targeted directed evolution and the suitability of BtR175 as a directed evolution target molecule for improving insecticidal activity.

## Materials and Methods

### Bacterial and insect strains

Wild-type T7 phage and Cry toxin-displaying phages were propagated and titrated in *Escherichia coli* BLT-gene10 as described previously (Ishikawa et al. 2007). *Escherichia coli* BLT-gene10 was made by transforming *E. coli* BLR (Novagen, Madison, WI) with a T7 capsid protein expression vector, pAR5615. *Escherichia coli* BL21 was used for the production of the wild-type or mutant Cry1Aa protoxin. Kinshu × Showa, a hybrid race of the silkworm, *B. mori* was reared on an artificial diet (Silk-mate; Nosan, Yokohama, Japan) at 25°C under a photoperiod of 16 h light 8 h dark.

### Construction of phage libraries of loop 2 mutants

Cry1Aa gene (GenBank accession: AAA22353) was cloned from *B. thuringiensis* subsp. *kurstaki* strain HD-1-Dipel as reported previously (Atsumi et al. 2005). To create phage libraries displaying loop 2-mutated Cry1Aa toxin, the regions encoding domains II–III of Cry1Aa toxin were amplified by polymerase chain reaction (PCR) using 5′-ttatcttcacctNNNNNNNNNNNNattaatacttggttc-3′ for 365–378 library, 5′-tcttcacctttatatNNNNNNNNNNNNcttggttcaggc-3′ for 367–370 library, 5′-gaagaattataNNNNNNNNNNNNccaaataatcagg-3′ for 371–374 library, 5′-ttatcttcacctNNNNNNNNNNNNgaactgtttgtc-3′ for 375–378 library, as sense primers and 5′-agctcattctcgagtgcggccgctctttctaaatcatattctgcctcaa-3′ as antisense primer. Next, the regions encoding domains I–III of Cry1Aa toxin were amplified by PCR using DNA fragments obtained by PCR as sense mega-primers and 5′-agaagtattaggtggggatccaatagaaactggttacaccccaa-3′ as antisense primer, digested and inserted into between BamHI and XhoI sites of T7Select 10-3b DNA (Novagen) as described previously (Ishikawa et al. 2007). The plaque-forming units of 365–368, 367–370, 371–374, and 375–378 libraries were estimated to be 1.38 × 10^6^, 1.31 × 10^6^, 1.28 × 10^6^, and 7.92 × 10^6^, respectively.

### Phage selection by biopanning and sequence analysis of displayed mutant toxins

A fusion protein of glutathione-*S*-transferase (GST) and a partial fragment (toxin-binding region, Glu1108–Val1464) of *B. mori* cadherin-like protein BtR175 (GST-BtR175-TBR) was produced using GST-tagged expression vector pGEX4t-3 (GE Healthcare, Chalfont, UK) as described previously (Fujii et al. 2013). In brief, DNA of BtR175-TBR was cloned into *Sma*I and *Not*I sites of pGEX4t-3. GST-BtR175-TBR was harvested as inclusion bodies consisting of protoxin and solubilized in 100 mmol L^−1^ Na_2_CO_3_, pH 9.5 containing 10 mmol L^−1^ dithiothreitol, and the buffer was changed into phosphate-buffered saline (PBS, 137 mmol L^−1^ sodium chloride, 2.7 mmol L^−1^ potassium chloride, 10 mmol L^−1^ sodium phosphate dibasic, 1.8 mmol L^−1^ potassium dihydrogen phosphate, pH 7.4). The GST-BtR175-TBR solution diluted 3.6 *μ*mol L^−1^ in PBS was incubated in the wells of an ELISA plate and washed three times with PBS, and then the plate was blocked with 2% bovine serum albumin (BSA). The wells were washed and preincubated with 150-*μ*L binding buffer (PBS with 2% BSA, 1% polyoxyethylene sorbitan monolaurate [Tween 20], and 1/5 (v/v) UV-inactivated phages) for 1 h at 37°C. The binding buffer (100 *μ*L) was replaced with phage solution (5 × 10^8^ pfu/100 *μ*L) diluted with SM buffer (0.1 mol L^−1^ sodium chloride, 8 mmol L^−1^ magnesium sulfate, 50 mmol L^−1^ Tris-HCl [pH 7.5], 0.01% gelatin) and the plate was incubated for 1 h at 37°C. Four wells were used per library. The wells were washed five times with PBST (PBS with 0.1% Tween) and bound phages were eluted with T7 elution buffer (PBS with 1% sodium dodecyl sulfate). Eluted phages were propagated by infecting *E. coli* BLT-gene10. The cycle consisting of biopanning and propagation of phage was repeated five times. Finally, 20–24 clones of phage were isolated from each well and 80–96 DNA sequences of the mutation-introduced region were determined.

### Preparation of mutant toxins

Mutant Cry1Aa toxins were prepared as described previously (Fujii et al. 2013). In brief, DNAs from mutant toxins displayed on the phage were inserted between *Spe*I and *Sac*I sites of GST-Cry1Aa protoxin fusion protein-expressing vector, pB9 and *E. coli* BL21 was transformed with each resulting vector. Mutant Cry1Aa toxins were harvested as inclusion bodies consisting of protoxin and solubilized in 100 mmol L^−1^ Na_2_CO_3_, pH 9.5 containing 10 mmol L^−1^ dithiothreitol, and then protoxin solutions were applied to a DEAE column (Shodex IEC DEAE-825; Showa Denko, Tokyo, Japan) connected to an HPLC system (Waters 600; Milford, MA), and protoxins were activated by 0.5 mg mL^−1^ trypsin in 20 mmol L^−1^ Tris-HCl, pH 8.3 containing 150 mmol L^−1^ NaCl for 2 h at 37°C in the column. Activated toxins were eluted using a linear gradient of 150–500 mmol L^−1^ sodium chloride and protein concentrations were determined by densitometry using AlphaDigiDoc™ (Alpha Innotech, San Leandro, CA). BSA was used as a standard.

### Binding kinetics analysis by surface plasmon resonance

Cry1Aa toxin-binding region of BtR175 (BtR175-TBR) was prepared as described previously (Fujii et al. 2013) and immobilized on a CM5 sensor chip using the amine-coupling method. Four different concentrations of mutant Cry toxins diluted in PBST (0.005% Tween, pH 7.4) were applied to the surface of the BtR175-TBR-immobilized CM5 sensor chip attached to Biacore J for 120 sec (GE Healthcare, Calfont, U.K.). For dissociation, toxin flow was replaced by PBST, and the response was recorded for 240 sec. The response curves were fit to a 1:1 Langmuir binding model using global fitting. Rate constants for association (*k*_a_) and dissociation (*k*_d_) were determined. The sensor chip was regenerated using 30 *μ*L of 10 mmol L^−1^ NaOH.

### Bioassay

Various concentration of activated mutant toxins were diluted with PBS, mixed with the artificial diet (2.5 g), and were placed in 9-cm Petri dishes. Third-instar larvae of *B. mori* were allowed to feed on the diet containing Cry1Aa inclusion body. After 24, 48, and 72 h, dead larvae were counted and significance of the mean differences was statistically analyzed by two-way ANOVA followed by Bonferroni multiple comparisons (Prism ver. 5; GraphPad, La Jolla, CA). The lethal concentration (LC_50_) was determined using probit analysis described by Bliss (1934). Twelve or 13 larvae were put on a petri dish and total 25 larvae were used for each toxin concentration. All experiments were done in triplicate.

### Cell toxicity assay using Sf9 cells expressing BtR175-TBR

Recombinant *Autographa californica* nucleopolyhedroviruses (AcNPVs) harboring BtR175-TBR were constructed using Bac MagicTM DNA Kits (Novagen), as described previously (Fujii et al. 2013). Sf9 cells were cultured at 28°C in Sf-900 SFM II (GIBCO BRL, Palo Alto, CA) containing 10% BSA. They were infected with AcNPV-BtR175-TBR. After 72 h, the cells were collected and seeded on a microcover glass. After adhesion, cells were washed in PBS to remove nonviable cells. The cover glass was placed on a two-hole slide glass (Matsunami, Osaka, Japan) filled with wild-type, ^371^WGLA^375^, and ^371^WPHH^375^ toxin solution. After 60 min of incubation at 25°C, the cells were observed under a phase-contrast microscopy. The total number of lowly refractile swelling cells out of 500 enhanced-GFP-expressing cells in five fields was counted. The experiment was repeated three times and the mean swollen cell rate (%) was calculated. Statistic analyses were conducted as described above.

## Results

### Selection of mutant toxins with higher binding affinities for BtR175-TBR

**Figure 1:**
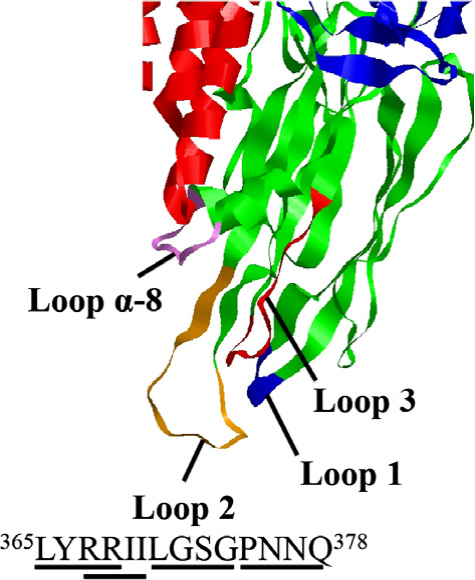
Mutation sites in Cry1Aa domain II loop 2. Each of the four underlined amino acids was randomly substituted to construct four phage libraries displaying mutant toxins.

Domain II loop 2, one of the BtR-binding regions of Cry1Aa, includes 14 amino acids and is located between residues 365 and 378 (Fig.1). Each of the 12 nucleotides corresponding to amino acid residues 365–368, 367–370, 371–374, or 375–378, respectively, were replaced with random nucleotides. These mutant toxins were displayed on T7 phage to create libraries. Biopanning was conducted using these four libraries with 2 × 10^9^ phages to select mutant toxins with higher BtR175-TBR-binding affinities than wild-type Cry1Aa. Eighty to 96 phages were screened from each library and the DNA sequences of the mutated regions of displayed Cry1Aa were determined. Theoretically, no identical clones can be expected in the 80–96 selected mutants, since four random serial substitutions generate 20^4^ variations. Nevertheless, many identical clones were obtained, as shown in Table1. According to the results of a previous study, one of the possible reasons for these concentrations of specific clones is a higher binding affinity between intermutant toxins and BtR175-TBR (Fujii et al. 2013). However, other factors, such as higher growth rates of certain phage clones, were also suggested.

**Table 1:** Frequency of mutant toxin-displaying phage clones concentrated from libraries by biopanning.

Clone names	Frequency
^365^VWGG^368^	11/92
^365^SPRS^368^	5/92
^365^GSYR^368^	5/92
^365^WYGA^368^	2/92
^365^VAQR^368^	2/92
^365^HAGG^368^	2/92
^367^PHGS^370^	6/84
^367^PRGG^370^	3/84
^367^NAGR^370^	3/84
^367^PRRA^370^	3/84
^367^RRTK^370^	3/84
^367^TNWP^370^	2/84
^367^HASQ^370^	2/84
^367^TRRR^370^	2/84
^371^WGLA^374^	22/96
^371^GHRR^374^	10/96
^371^RGPR^374^	8/96
^371^WRPQ^374^	7/96
^371^SVRR^374^	6/96
^371^WPHH^374^	4/96
^371^PHRP^374^	3/96
^371^QSRA^374^	3/96
^371^ADPL^374^	3/96
^371^GMRA^374^	2/96
^371^QERE^374^	2/96
^371^PASD^374^	2/96
^371^RVRP^374^	2/96
^375^VLRG^378^	3/80
^375^RPRL^378^	2/80
^375^ARGR^378^	2/80
^375^DPRA^378^	2/80
^375^VPPR^378^	2/80

### Binding affinities between mutant toxins and BtR175-TBR

**Figure 2:**
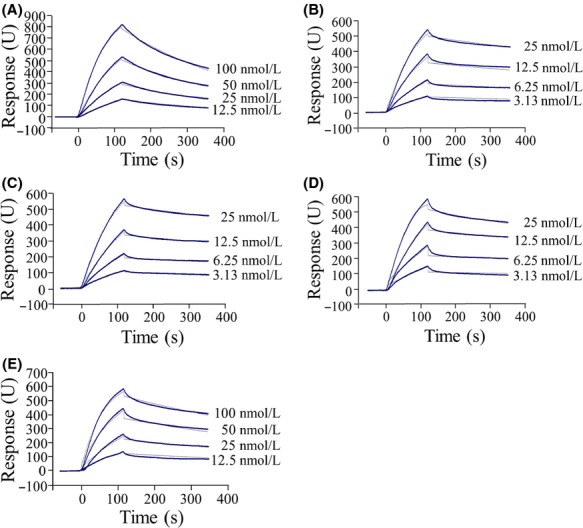
Biacore sensorgrams for the binding of wild-type and mutant Cry1Aa toxins to BtR175-TBR. Actual response curves are shown by thick lines and the 1:1 Langmuir fit is indicated by thin lines: (A) wild type, (B) ^371^WGLA^374^, (C) ^371^WPHH^374^, (D) ^371^WRPQ^374^, (E) ^371^PHRP^374^.

Attempts were made to express recombinant mutant toxins, including ^365^VWGG^368^, ^367^PHGS^370^, ^371^GHRR^374^, ^371^RGPR^374^, ^371^SVRR^374^, ^371^WGLA^374^, ^371^WPHH^374^, ^371^WRPQ^374^, and ^371^PHRP^374^, in *E. coli*. The recombinant toxins were activated by trypsin and purified. Toxin productions of ^365^VWGG^368^ and ^367^PHGS^370^ in *E. coli* as inclusion bodies were insufficient to further analysis. Three mutant toxins, ^371^GHRR^374^, ^371^RGPR^374^, and ^371^SVRR^374^ were trypsin sensitive. As we previously reported, mutant toxins produced by *E. coli* did not have trypsin tolerance frequently (Ishikawa et al. 2007). Only four mutant toxins, ^371^WGLA^374^, ^371^WPHH^374^, ^371^WRPQ^374^, and ^371^PHRP^374^, were trypsin tolerant (Fig. S1) and obtained in sufficient quantity for analysis. BtR175-TBR-binding affinities were determined using surface plasmon resonance (SPR) (Fig.2). The Langmuir 1:1 binding model was used to calculate the association rate constant (*k*_a_ [mol L^−1^ sec^−1^]) and dissociation rate constant (*k*_d_ [sec^−1^]). The dissociation constant (*K*_D_ [mol L^−1^]), the primary parameter for binding affinity, was then calculated according to the formula, *K*_D_ = *k*_d_*/k*_a_. The *K*_D_ values of ^371^WGLA^374^, ^371^WPHH^374^, ^371^WRPQ^374^, and ^371^PHRP^374^ were 16-, 16-, 50-, and threefold, respectively, lower than that of the wild-type toxin (Table2). Thus, ^371^WGLA^374^, ^371^WPHH^374^, and ^371^WRPQ^374^ should exhibit higher binding affinities for BtR175-TBR than the wild-type toxin.

**Table 2:** BtR175-binding affinity parameters of the wild-type and mutant toxins obtained using the 1:1 Langmuir-binding model shown in Figure3.

Clone names	*k*_a_ (×10^5^ mol L^−1^ s^−1^)	*k*_d_ (×10^−3^ s^−1^)	*K*_D_ (×10^−8^ mol L^−1^)
Wild type	1.06	2.67	2.50
^371^WGLA^374^	3.90	0.61	0.156
^371^WPHH^374^	4.23	0.66	0.155
^371^WRPQ^374^	20.5	1.02	0.05
^371^PHRP^374^	1.48	1.22	0.83

### Insecticidal activities of mutant toxins with high BtR175-TBR-binding affinities

**Figure 3:**
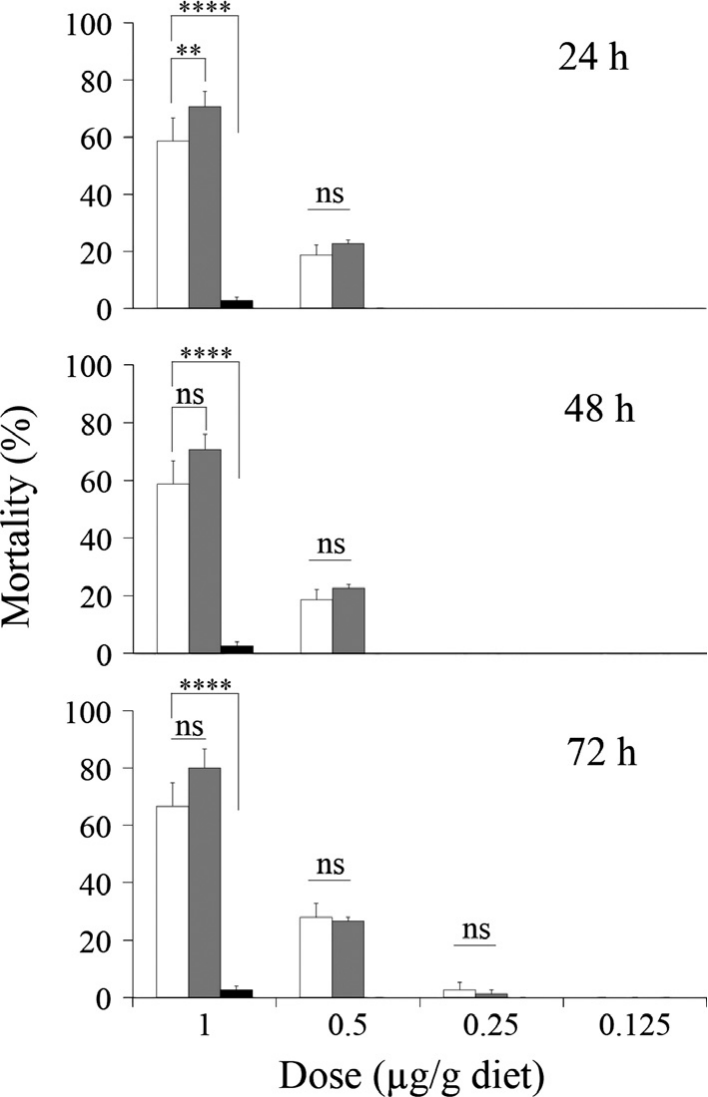
Insecticidal activities of wild-type and high-BtR175-TBR-binding-affinity mutant toxins. Seventy-five third-instar larvae were assessed in every four toxin concentration. Dead larvae were counted after 24 h, 48 h, and 72 h. The data shown are the means calculated from three independent experiments. Bars represent the corresponding standard errors. White, wild type; gray, ^371^WGLA^374^; black, ^371^WPHH^374^. Statistical analyses were performed by using two-way ANOVA before Bonferroni multiple comparisons. “ns” indicates no significant difference; an asterisk indicates a significant difference (**P < 0.01; ****P < 0.0001).

Insecticidal activities against *B. mori* were determined for ^371^WGLA^374^, ^371^WPHH^374^, and ^371^WRPQ^374^ toxins. The ^371^WGLA^374^ mutant exhibited a slightly higher insecticidal activity than the wild-type toxin, but no significant differences were observed in six of the seven experimental conditions (Fig.3). The ^371^WPHH^374^ mutant showed significantly lower activity (Fig.3). The ^371^WRPQ^374^ mutant was unable to kill larvae at doses of 1 *μ*g/g diet in a preliminary experiment (data not shown). The LC_50_ value of ^371^WGLA^374^ 48 h after administration was 1.15-fold lower than that of the wild-type toxin (Table3). Therefore, no evidence for enhanced insecticidal activity resulting from enhancement of the toxin-binding affinity for BtR175-TBR was observed.

**Table 3:** LC_50_ and relative toxicities of wild-type and ^371^WGLA^374^ mutant toxins to *B. mori* third-instar larvae at 48 h after administration.

	Wild-type	^371^WGLA^374^
LC_50_ (*μ*g/g diet)	0.864	0.752
(95% CI)	(0.796–1.004)	(0.680–0.843)
Relative toxicity^1^	1.00	1.15

LC, lethal concentration.

1 LC_50_ of wild-type/LC_50_ of mutant.

### Cytotoxicity of mutant toxins to BtR175-TBR-expressing Sf9 cells

**Figure 4:**
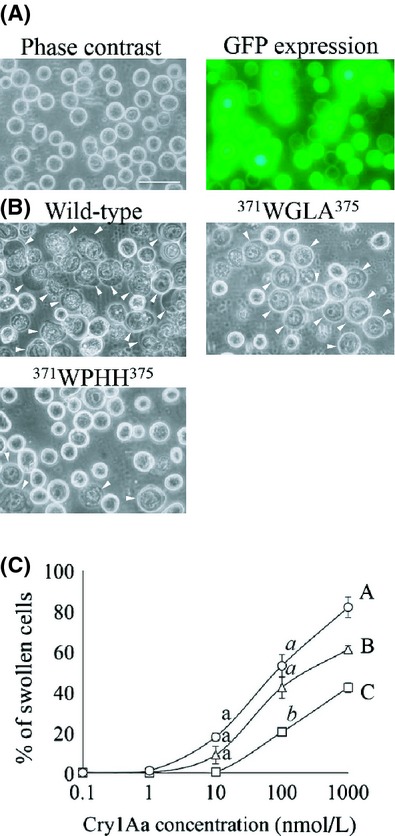
Cytotoxicities of wild-type, ^371^WGLA^375^, and ^371^WPHH^375^ toxins against BtR175-TBR-expressing Sf9 cells. (A) BtR175-TBR-expressing Sf9 cells incubated with no toxin (left) and their GFP expression (right) as a BtR175-TBR-coexpression marker. The scale bar indicates 50 *μ*m. (B) Cell swelling of BtR175-TBR-expressing Sf9 cells incubated with 100 nmol L^−1^ wild-type, ^371^WGLA^375^, and ^371^WPHH^375^ toxins for 1 h. Arrowheads indicate low refractile swelling cells. (C) Swollen cell ratios are given for BtR175-TBR-expressing cells in response to wild-type (○), ^371^WGLA^375^ (▵), and ^371^WPHH^375^ (□) toxins. Error bars represent the standard error of the mean but some are too small to see on the scale used. Statistic analyses were performed by using two-way ANOVA before Bonferroni multiple comparisons. Dots labeled with the same letters indicate no significant difference (*P* > 0.05).

The introduced mutations may have affected the mode of action of the Cry toxin outside of the cadherin-binding phase. To evaluate the effects of only altering the BtR175-TBR-binding affinity, we investigated the cytotoxicity of ^371^WGLA^374^ and ^371^WPHH^374^ using BtR175-TBR-expressing Sf9 cells. Sf9 cells coexpressing BtR175-TBR and ABCC2 exhibit very high susceptibility (∼100 pmol/L) to the toxin, probably because of synergistic effects between both receptors (Tanaka et al. 2013). In contrast, Sf9 cells expressing only BtR175-TBR showed low toxin susceptibility (∼100 nmol/L). This single receptor expression system was used to evaluate improvements in mutant toxin BtR175-TBR-binding affinity. The Sf9 cells had been previously infected with BtR175-TBR harboring AcNPV for 72 h. Coexpressed GFP showed that more than 80% of the Sf9 cells were infected with the virus and seemed to express BtR175-TBR (Fig.4A). Subsequently, cells were incubated with each respective toxin for 1 h. In accordance with a previously reported method (Tanaka et al. 2013), low refractile swelling cells as shown in Figure4B were used to calculate a cell swelling rate (as susceptibility cell rate) in Figure4C. Cytotoxicity curves suggest that the activities of the two mutant toxins were statistically the same or lower than that of wild-type Cry1Aa (Fig.4B and C) in cells expressing only BtR175-TBR.

## Discussion

### Enhancement of inter-Cry1Aa toxin-BtR175-TBR affinity by introducing mutations in domain II loop 2

In antibody protein engineering by directed evolution, mutant antibodies with high antigen-binding affinities have been successfully generated by introducing mutations into the variable loop regions that bind to the antigen (Schier et al. 1996; Thompson et al. 1996). In the present study, random sequences of four amino acids were substituted into the four regions of Cry1Aa toxin domain II loop 2. Mutant toxins with higher binding affinities for BtR175 were screened by panning. As a result, several phage clones were confirmed in the 371–374 library (Table1) and three phages displayed mutant toxins with BtR175-TBR-binding affinities that were 16-, 16-, and 50-fold higher than that of the wild-type toxin (Table2). The degree of affinity maturation of the loop 2 mutants was equal to or greater than that of loop 3 mutants (Fujii et al. 2013). These results suggest that both loops 2 and 3 contain important regions for binding BtR175. This conclusion also agrees with the previously reported hypothesis that Cry1Aa binds to cadherin-like receptors at multiple sites presented by several loops (Fujii et al. 2012). The current study demonstrates that loop 2 is one of the most effective regions for introducing Cry1Aa affinity maturation to cadherin-like receptors for the purpose of directed evolution of the toxin.

### Low effect on insecticidal activity of Cry1Aa affinity maturation to BtR175-TBR

Reports of Cry1A-resistant insect strains with mutations in their cadherin-like receptors (Morin et al. 2003; Yang et al. 2009; Gahan et al. 2010) suggest that the cadherin-like receptor plays an important role in the insecticidal activity of Cry1A. Therefore, increasing the Cry toxin-binding affinity for BtR175-TBR by directed evolution was expected to result in more active toxins. However, despite 16- or 50-fold higher binding affinities of selected mutant toxins, correspondingly enhanced activity was not observed in *B. mori* larvae (Fig.3, Table3) or in Sf9 cells expressing BtR175-TBR (Fig.4). Some papers have indicated that Cry1Aa binding to cadherin-like receptor does not correlate with insecticidal activity (Pigott et al. 2008; You et al. 2008). Even when the cadherin-like receptor was completely functional, mutations in ABCC2 genes resulted in a high resistance to Cry1A (Baxter et al. 2008, 2011; Franklin et al. 2009; Atsumi et al. 2012). In addition, *B. mori* ABCC2 (BmABCC2) conferred higher susceptibility in Sf9 cells compared to BtR175 (Tanaka et al. 2013). These conflicting studies indicate that the function and importance of the cadherin-like receptor in Cry1A toxicity is still poorly understood. Further studies are needed to explain why even a greater than 10-fold higher affinity for the cadherin-like receptor does not result in higher insecticidal activity.

As noted above, loop 3 mutations might affect other phases that occur during pore formation, resulting in no enhancement in toxicity (Fujii et al. 2013). The same can be thought of with regard to loop 2. Loop 2 mutations might interfere with any of the other interactions between Cry1Aa and other receptors, including APN, ALP, and ABCC2.

### Targets for affinity maturation of the Cry toxin

In this study, Cry1Aa activity to *B. mori* was not enhanced, although we achieved a 16- to 50-fold increase in the binding affinity of Cry1Aa to BtR175. By the way, wild-type Cry1Aa is originally highly toxic to *B. mori*, and binds to BtR175 with high affinity. Consequently, under this experimental condition, improving the affinity of Cry toxin for the BtR175-TBR might be difficult to result in enhancement of insecticidal activity beyond the original level. Nevertheless, as described above, Cry toxin binding to cadherin-like receptors should be of great significance in the exertion of toxicity. Directed evolution of a Cry toxin targeting cadherin-like receptors should be useful for developing insecticidal activity in cases when no native affinity exists. For example, *Tenebrio molitor* larvae are susceptible to Cry3Aa but not to Cry1Aa. In our experiment, *T. molitor* cadherin-like protein binds to Cry3Aa but not to Cry1Aa (data not shown). If the nonbinding of Cry1Aa to the cadherin-like protein determines the nonsusceptibility of *T. molitor* larvae, a mutant toxin that binds *T. molitor* cadherin-like protein might be active in *T. molitor* larvae. Based on this hypothesis, the direct evolution of Cry1Aa to *T. molitor* cadherin-like protein is now ongoing.

Receptors play important roles in the exertion of the insecticidal activity of Cry toxins. Our strategy, involving the evolutionary molecular engineering of Cry toxin, is expected to yield major artificial improvements in the toxicity or specificity of Cry toxins.

The English in this document has been checked by at least two professional editors, both native speakers of English. For a certificate, please see http://www.textcheck.com/certificate/oQSHu0.

## Supporting Information

Figure S1 SDS-PAGE electrophoretogram of trypsin-activated wild-type toxin, ^371^WGLA^375^, and ^371^WPHH^375^. Protoxins were activated by 0.5 mg^−1^ mL^−1^ trypsin in 20 mmol L^−1^ Tris-HCl, pH 8.3 containing 150 mmol L^−1^ NaCl for 2 h at 37°C in the DEAE column. Lane 1: wild-type toxin, lane 2: ^371^WGLA^375^, lane 3: ^371^WPHH^375^.
