# Supplementary figures and images for: Affinity maturation of Cry1Aa toxin to the Bombyx mori cadherin-like receptor by directed evolution based on phage display and biopanning selections of domain II loop 2 mutant toxins
Source: Microbiologyopen. 2014 Jul 16;3(4):568–77. doi: 10.1002/mbo3.188 (PMC4287183; doi:10.1002/mbo3.188)

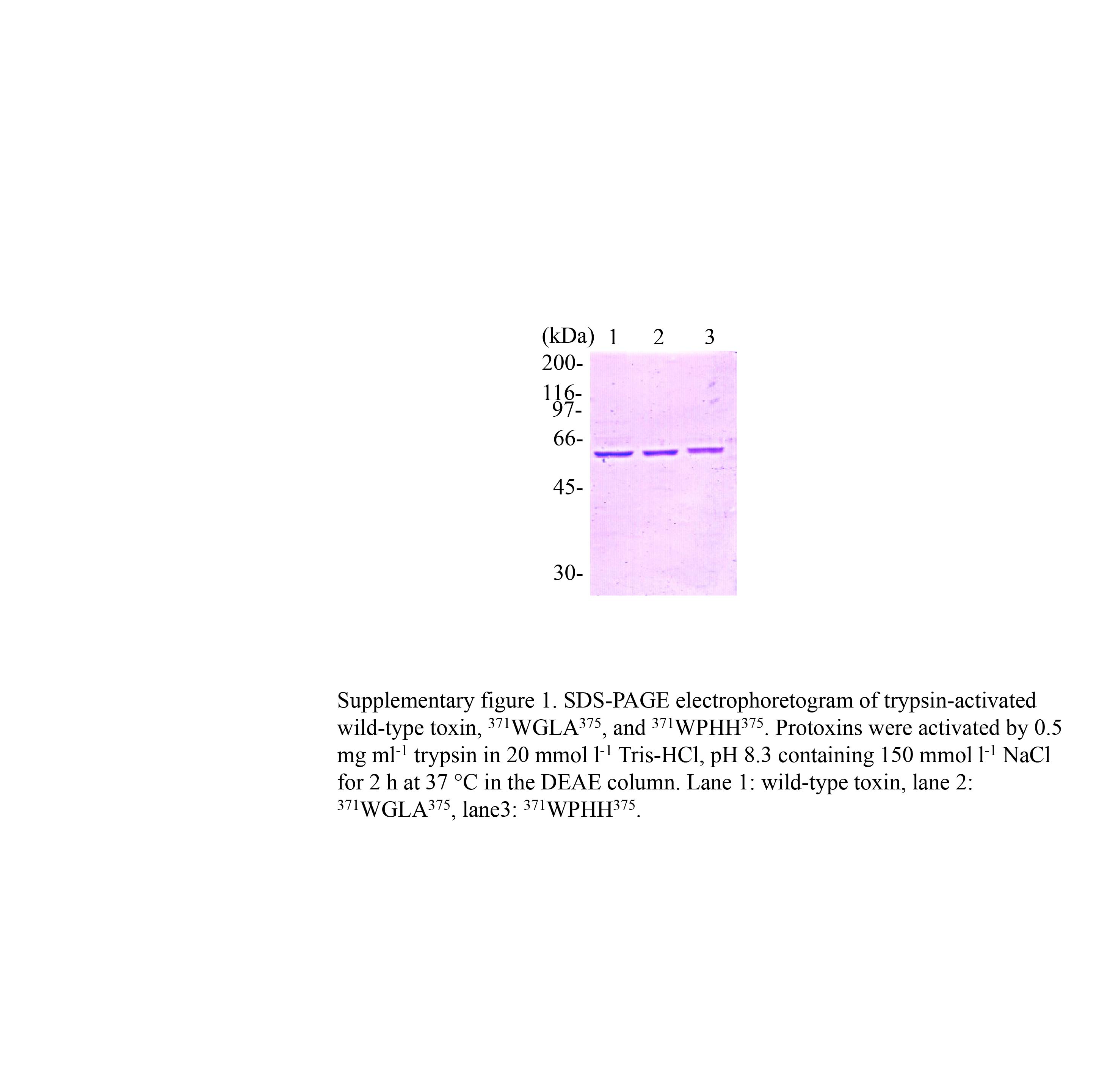

Supplement: Figure S1 — SDS-PAGE electrophoretogram of trypsin-activated wild-type toxin, 371WGLA375, and 371WPHH375. Protoxins were activated by 0.5 mg−1 mL−1 trypsin in 20 mmol L−1 Tris-HCl, pH 8.3 containing 150 mmol L−1 NaCl for 2 h at 37°C in the DEAE column. Lane 1: wild-type toxin, lane 2: 371WGLA375, lane 3: 371WPHH375. [file mbo30003-0568-sd1.jpg]
